# Why Do Nurses Work While Sick? An Exploratory Study of Nurse Leaders' Cognitive Preferences Toward Presenteeism

**DOI:** 10.1155/2024/5522654

**Published:** 2024-11-08

**Authors:** Wenzhen Li, Wei Wang, Geyan Shan, Hongxia Wang, Shujie Guo, Yongxin Li

**Affiliations:** ^1^Institute of Psychology and Behavior, Henan University, Kaifeng, China; ^2^Business School, Henan University, Kaifeng, China; ^3^Department of Outpatient, Henan Province Hospital of TCM, Zhengzhou, China; ^4^Department of Outpatient, Henan Provincial People's Hospital, People's Hospital of Zhengzhou University, Zhengzhou, China

**Keywords:** cognition, head nurses, nurses, presenteeism

## Abstract

**Purpose:** Nursing is a representative profession with a high prevalence of presenteeism, which is usually associated with negative outcomes. Therefore, it is important to explore the antecedent factors of nurse presenteeism behavior. This study aims to explore the impact and mechanism of head nurses' cognitive preference toward presenteeism on subordinate nurses' presenteeism (SNP), and the mediation effects of subordinate nurses' perception of head nurses' cognitive preference.

**Patient and Methods:** A cross-sectional study was conducted from July to August 2022. A total of 256 head nurses and 1424 subordinate nurses were recruited from six hospitals located in Zhengzhou, Henan Province, China. The Nurse Presenteeism Questionnaire (NPQ) and Cognitive Preference Questionnaire were used to assess head nurses' cognitive preference toward presenteeism, SNP, and subordinate nurses' perception of head nurse's cognition of presenteeism. We conducted description, multilevel correlation, and multilevel structural equation models for the data analysis.

**Results:** In the past 6 months, 93.4% of nurses experienced presenteeism. Within and between the team, head nurses' cognitive preference toward presenteeism is positively associated with SNP. The mediation effect of nurses' perception of head nurses' cognitive preference was also significant.

**Conclusion:** Head nurses' cognitive preferences are essential predictors of subordinates' presenteeism. In this process, subordinates' subjective initiative plays a crucial role.

**Implications for Nursing Management:** Hospital managers should focus on head nurses' values to formulate multiple interventions of presenteeism and strengthen communication between leaders and subordinates to promote transforming negative outcomes into positive outcomes.

## 1. Introduction

Presenteeism is defined as an individuals' behavior of attending work despite complaints and ill-health that should prompt absence from work and rest [1]. Nurses are a characteristic occupational group with a high incidence of presenteeism [1]. For instance, the incidence of presenteeism among Spanish medical staff was 53% [2] and 91.4% in Portuguese nurses [3]. In China, the prevalence of presenteeism among nurses was 94.25% [4]. High presenteeism among nurses may have severe consequences such as physical and psychological health problems, decrease in care quality, increase in burnout, and ultimately, damage to the social economy [5].

Considering the extensive negative consequences of the prevailing incidence of nurses' presenteeism, the predictive factors were explored. Rainbow and Steege used a theoretical model and showed that an imbalance of work life, stressful work environment, poor health and well-being, and professional nursing identity were the main predictors of presenteeism in nursing [6]. However, within the work environment, interpersonal interaction with employees is a non-negligible factor affecting presenteeism [7–10]. Accordingly, we focused on head nurses for their critical roles. Head nurses' presenteeism promotes the incidence of followers' presenteeism, and leader identification enhances this relationship [11]. Moreover, Shan and colleagues argued that authoritarian leadership increases nurses' presenteeism through their workload [9].

Nevertheless, head nurses' presenteeism is a behavioral choice based on their own cognition of presenteeism, as an individual receiving external information, subsequently transforming it into internal psychological activities, thereby controlling their behavior [12]. Generally, previous research generally examined the effect of head nurses' external behaviors; however, few focused on the association between head nurses' individual cognition and subordinates' presenteeism [13, 14]. Therefore, this study aimed to explore head nurses' influence of cognitive preference for presenteeism (HCP) on subordinate nurses' presenteeism (SNP) to explore the internal mechanism of top-down influence within the organization.

In addition, subordinate nurses' perception of head nurses' cognition of presenteeism (SNPH) could serve as a mediator between the HCP and SNP relationship. Since SNPH could reflect the HCP directly, HCP is the basis and prerequisite for presenting and interpreting work environments and organizational information, which further impacts subordinates' presenteeism [15–17].

In summary, the main aim of the current study was to explore the antecedents and mechanisms of nurses' presenteeism. It specifically focused on the effect of HCP on SNP, contributing to the effective implementation of nursing management and intervention strategies for nurses' presenteeism.

## 2. Head Nurses' Cognitive Preference Toward Presenteeism and SNP

The head nurse, as the direct supervisor of nurses, has an important impact on nurses' work behaviors [4, 11]. Leaders affect employee health through various pathways [18]. One is climate control and identity management, which entails leaders creating a group climate and social identity within a team by cultivating and crafting shared opinions and actions [19]. Another potential pathway is modeling, which suggests that when leaders act as role models, their health behaviors, states, and attitudes impact followers' health behaviors [16, 18, 20].

Based on their cognition, leaders establish the organizational climate of presenteeism by dominating the work environment and nourishing common values, thus affecting the occurrence of juniors' behaviors. Leaders dominate the organizational climate [20–22]. In this context, Schein interestingly pointed out that organizations do not form automatically or accidentally but are established when someone takes a leadership role. Leaders elucidate and reinforce their values to form an organizational climate through culture-embedding mechanisms [23, 24]. Organizational climate significantly impacts employees' work behaviors [25–28]. For instance, Kuenzi and colleagues claimed that positive organizational climates could promote employees' ability to cope with complex work environments and enhance their work efficiency and creativity [27].

In presenteeism, a qualitative study confirmed that upper organizational management implies the legitimacy of sickness absenteeism and presenteeism within the organization based on their attitudes toward presenteeism. Employees perceive presenteeism values from upper management and shape an organizationally attitudinal consensus, thereby impacting the occurrence of individual presenteeism [5, 15]. In addition, Dietz and colleagues stated that leaders' specific attitudes and behaviors would reinforce organizational climates and strengthen the influence of team culture on employee presenteeism [16].

In the nursing group, nurses tend to describe their workplace as a “sanctuary,” and the relationship with their team as “family.” [7, 29] Nurses tend to have a high attachment, intense reliance, and a remarkable identity in the workplace. Therefore, SNP is exceptionally susceptible to organizational climate. Furthermore, the various organizational climates formed by head nurses have prompted various SNP.

The other potential pathway is role modeling, namely, leaders are role models for employees. Leaders' attitudes and cognition toward presenteeism impact their behaviors, as individual cognition dominates individual behavior [30]. Specifically, individuals who regard presenteeism as positive prefer to present while sick. In contrast, individuals who perceive presenteeism as negative would be inclined to be absent while sick. According to social learning theory, within a team, leaders are the role models, as they have a particular status in the work environment [31–33]. The subordinate will observe and imitate leaders' work behavior in the state of ill-health (absent or present), which is controlled by leaders' cognition. Therefore, based on the relationship between cognition and social learning theory, leaders' values, cognition, and belief may impact subordinates' behavior.

In nursing groups, head nurses serve the dual roles of caregivers and leaders. Head nurses provide the organizational information prior to the conveyor and role model of professional nursing skills, who master resources and authority [9]. Consequently, HCP probably serves as social information in a nursing setup that affects SNP. In addition, within teams, various SNP fluctuates when social information about presenteeism is obtained from head nurses.

In summary, we argue that head nurses impact SNP through shaping organizational climates and role modeling, that is, cultivating common attitudes consistent with their presenteeism values or providing social information on cognitive references to followers. Therefore, we hypothesized as follows.

**Hypothesis 1 .**

HCP is positively associated with SNP.

### 2.1. Mediation Effect of SNPH

This implies that SNPH is involved in the above two potential pathways, that is, in the process of HCP impact SNP. Perception refers to organizing, identifying, and interpreting sensory information to represent and understand presented information or context [34]. In shaping organizational climates, only subordinate nurses specifically perceived head nurses' cognition toward presenteeism, and aggregated, generated, and formed as organizational attitude consensus, thereby affecting subordinates' presenteeism [15].

Furthermore, in the role-modeling pathway, according to social information processing theory [35], the social environment provides cues regarding expectations and norms concerning individual attitudes and behaviors, which are adopted to construct and interpret events. Individuals attain cues and adopt their attitudes and cognitive evaluation through observation. Leaders' attitudes, expectations and cognitive preferences toward presenteeism are the social cues within the organization, as leaders possess more authority. Only when subordinate nurses observe, perceive and recognize these social cues could the social cues change subordinates' attitudes and preferences toward presenteeism, thereby impacting their own behaviors. In addition, Dietz and colleagues stated that only if subordinate nurses observe, perceive and obtain HCP as a social information cue can they adopt their own work behaviors, i.e., hindering or importing presenteeism [16]. Therefore, we hypothesized as follows:

**Hypothesis 2 .**

HCP via SNPH is positively related to SNP.

**Figure 1:**
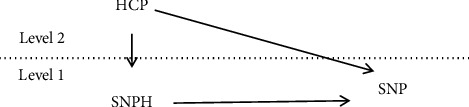
Theoretical model.

As outlined above, to explore the influence of a direct leader on SNP, the current study examined the direct cross-level effects of HCP on SNP. We also examined the mediating effects of SNPH. The integrated conceptual model is illustrated in Figure 1.

## 3. Theoretical Model

### 3.1. Materials and Methods

#### 3.1.1. Procedure and Participants

We adopted a cross-sectional questionnaire survey, which recruited 256 head nurses and 1424 junior nurses from six hospitals located in Henan Province, China, using convenience sampling. The study was conducted between July and August 2022. Before the survey, the research group communicated with the nursing management of the target hospitals to intimate them about the study's purpose and research plan and obtained their permission. To ensure a high match between the head nurses and subordinate nurses, two trained research assistants visited each department of the sampling hospitals and distributed digital and paper questionnaires to the head nurses of the department and to more than three subordinate nurses. The digital and paper questionnaires were bound and coded. Before completing the questionnaires, all participants were informed of the study's purpose and provided oral informed consent. The surveys were conducted anonymously. Finally, the researchers retrieved the completed questionnaires filled in by the participants, checking the data quality. The Ethical Review Board of the Institution of Psychology and Behavior, Henan University approved the study design.

#### 3.1.2. Measures

General demographic characteristics, including sex, age, tenure, marital status, educational level, and technical title, were collected from the participants. To ensure the accuracy of matching data, head nurses were asked to fill in the last four digits of their mobile numbers, and nurses were asked to fill in the last four digits of their head nurses' mobile numbers. Age and tenure were analyzed as continuous variables. Data on other types of information were collected using multiple choice questions. Participants' sex comprised males and females, and marital status was categorized as unmarried, married, or other. Educational level data were collected using multiple choice, which comprised senior high school or technical secondary school, junior college, bachelor's degree, and master's degree and above. Technical titles included nurses, nurse practitioners, nurse-in-charge, deputy chief nurses, and chief nurses.

In this study, SNP was evaluated by subordinate nurses using the Nurse Presenteeism Questionnaire (NPQ) developed by Shan and colleagues [36]. The NPQ contains 11 items. Participants were required to recall the frequency of experiences in which they had attended work in the past 6 months despite feeling sick. Each item was rated on a four-point scale (0: *never*; 1: *once*; 2: *2–5 times*; 3: *more than 5 times*) without reverse scoring. High scores indicated a frequent incidence of presenteeism. In the current research, Cronbach's *α* of the SNPQ was 0.94.

Furthermore, HCP was assessed by head nurses using a Cognitive Preference Questionnaire [13]. The questionnaire formulated a scenario in which a nurse *(Xiaowang*) had a cough, runny nose, and complained of a slight fever in the morning. The questionnaire comprised six items divided into three dimensions: anticipation preference (one item), benefit preference (four items), and management preference (one item). The head nurses were required to answer questions from their own perspectives. All the questions were dichotomous. Regarding anticipation preference, *if you were the direct leader (head nurse) of Xiaowang, you would suppose Xiaowang should be (1) attending work while ill*, *or (2) resting at home.* The first answer of all items was recorded as “1,” and the second answer as “0.” Moreover, referring to benefit preference, the participant had to answer the following question: *“if Xiaowang attends work while ill, from the current/long-term perspective*: *in your view, Xiaowang' s behavior toward Xiaowang/organization is (1) beneficial, or (2) detrimental.”* The first answer of all items was scored as “1,” and the second answer as “0.” Regarding management preferences, *from the supervisor's perspective, the participant answered the question: “if Xiaowang attends work while ill, you would (1) praise her, (2) pay attention silently, and (3) advise her to rest at home.*” The first and second answer was scored as “1,” and the third answer as “0.” The total average score of the six items represents the HCP score and higher scores indicate positive attitudes toward presenteeism.

The SNPH was also measured by subordinate nurses using a Cognitive Preference Questionnaire [13]. Contrastingly, subordinate nurses were required to answer questions from *Xiaowang's* perspective and evaluate their head nurses' attitudes toward presenteeism. All the questions were dichotomous. For example, if *you were Xiaowang, you would suppose that your direct leader (head nurse) would (1) hope you turn up for work, or (2) hope you rest at home.* The former answer was recorded as “1,” and the later answer was recorded as “0.” The total average score of the six items indicates SNPH. Higher scores indicated head nurses' positive attitudes toward presenteeism, as perceived by subordinate nurses.

#### 3.1.3. Statistical Analysis

The Statistical Package for the SPSS Version 23.0 and Mplus were used for data sorting and analysis. First, presenteeism among junior nurses was described using absolute and relative frequencies. Second, a *t*-test or chi-square test was used to assess junior nurses' presenteeism in relation to demographic variables with a 95% confidence interval.

Finally, to test the hypotheses, a multilevel mediation model with 2-1-1 was designed, as our data had a nested structure. Specifically, subordinate nurses (Level 1) were nested within teams (Level 2), and HCP was a team-level variable. Therefore, whether a multilevel model existed in our data was tested. For this purpose, ICC (1) values were calculated [37]. The results showed that the ICC (1) value of SNP was 0.155(> 0.059), and the ICC (1) value of SNPH was 0.157(> 0.059). Thus, it is appropriate that our data be analyzed as a multilevel model [38]. Subsequently, a multilevel mediation model was used to examine the effect of the Level 2 variable (HCP) on the Level 1 mediator (SNPH), which was further associated with the Level 1 outcome (SNP). In addition, the model presumes that the a1 and a2 paths are equal and differentiate between the within-team indirect effect (ab1) and the between-team indirect effect (ab2) [39]. Both the within-team and between-team indirect effects aroused our interest. We suppose that the within-team effect indicates that the head nurse, as a role model, provides attitude cues about presenteeism and influences followers' presenteeism. Furthermore, the between-team indirect effect indicates that head nurses' attitudes and cognition toward presenteeism shape followers' common opinions and establish an organizational climate, thus influencing followers' presenteeism. Consequently, two indirect effects have been reported. The model defines the variables measured at the team level as between-team variables (HCP). To isolate the within-team direct effect and between-team direct effect, the average of the team aggregate of SNPH (${\overset{¯}{M}}_{j}$) was added as a between-team variable (Level 2) [40]. Furthermore, ${\overset{¯}{M}}_{j}$ represents the organizational climate of the group. Variables were measured at the junior nurses' level as within-team variables (Level 1): SNPH and SNP. Moreover, the group mean center was used to define mediators at the team level [41]. The MLR estimator was used for this study because not all variables were normally distributed. [42].

## 4. Result

### 4.1. Demographic Characteristics of Participants

After data sorting, 1572 valid questionnaires from 233 nursing teams, including 233 head nurses and their 1339 subordinate nurses, were included in the analysis. The effective response rate was 93.5%. Referring to head nurses, 229 female (98.3%) and 4 male (1.7%) participants were included. The head nurses' average age was 42.19 years (SD = 5.76), and their average tenure was 22.06 years (SD = 6.77). In addition, 226 (97.0%) were married, four (1.7%) were unmarried, and three (1.3%) responded with “others.” Regarding educational level, 214 (91.8%) had a bachelor's degree, and 19 (8.2%) had a master's degree or higher. Among the head nurses, 54.1% (126) had the title of nurse-in-charge or lower, and 45.9% (107) had the title of deputy chief nurse or higher. Among junior nurses, 48 (3.6%) were male and 1291(96.4%) were female. The average age of junior nurses was 31.83 years (SD = 5.36), and the average tenure was 9.76 years (SD = 5.97). A total of 405 (30.2%) junior nurses were married and 934 (69.8%) were unmarried. Furthermore, 94 (7.0%) had a junior college education or below, 1199 (89.5%) had a bachelor's degree, and 46 (3.4%) had a master's degree or higher. Regarding job technical titles, 110 (8.2%) held the title of nurse, 465 (34.7%) held the title of nurse practitioner, 753 (56.2%) held the title of nurse-in-charge, and 11(0.8) held the title of deputy chief nurse. The number of subordinate nurses included in the teams ranged from 3 to 22.

### 4.2. Applicability of Research Data

Considering that two of the three variables involved in the current study were measured by junior nurses, the common method bias was analyzed by controlling for the effects of an unmeasured latent method factor test [42]. Compared to the original two-factor model (CFI = 0.911; TLI = 0.898; SRMR = 0.030; RMSEA = 0.063), the CFI and TLI of the ULMC model with the added method factor were 0.958 and 0.944, respectively, which increased by 0.047 (< 0.1) and 0.046 (< 0.1), respectively. Furthermore, SRMR and RMSEA decreased by 0.024 (< 0.05) and 0.022 (< 0.05), respectively. Therefore, serious common method biases were not observed in the current research [43].

### 4.3. SNP and Differences in Demographic Characteristics

The mean SNP was 1.32 ± 0.84. In the past six months, 1250 (93.4%) subordinate nurses had experienced presenteeism at least once. The differences in junior nurses' NPQ scores according to demographic characteristics were not significant.

### 4.4. Correlation Analysis of Research Variables

As shown in Table 1, SNPH was significantly and positively correlated with the SNP. Furthermore, HCP was significantly positively associated with SNPH but not with SNP.

### 4.5. Results of the Multilevel Path Model

**Figure 2:**
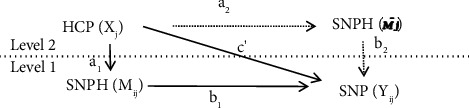
Design of the 2-1-1 model. Note: The design of the cross-cluster-level mediation model with 2-1-1 is given in solid and dotted lines. *X*_*j*_ represents head nurses for a given team *j*; *M*_*ij*_ means SNPH; *Y*_*ij*_ represents SNP, for subordinate nurse *i* cluster *j*. *M*_*j*_ means the average of the team aggregate of SNPH. The a1 and a2 paths are presumed to be equal in this model.

**Figure 3:**
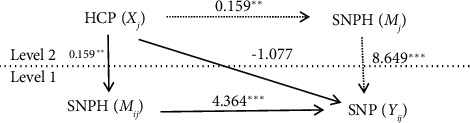
Results from a multilevel path model.

To test these hypotheses, an overall multilevel path model was analyzed. The results of the path model are shown in Figures 2 and 3 and Table 2. The results found no significant direct effect of HCP on SNP (γ = −1.077; *p*=0.332; 95% CI: [−3.254, 1.101]). Hence, Hypothesis 1 was not supported. Moreover, at the within-team level, HCP had a significant positive indirect effect on SNP via SNPH (γ = 0.692; *p*=0.009; 95% CI: [0.174, 1.210]). In addition, at the between-team level, the HCP positive impact on SNP through SNPH was also significant (γ = 1.372; *p*=0.008; CI 95%: [0.364, 2.379]). Thus, Hypothesis 2 was supported.

## 5. Discussion

### 5.1. General Discussion

The high prevalence of presenteeism among nurses is related to subsequent negative consequences [4]. Head nurses are essential predictors since they are the direct leaders of junior nurses, communicating frequently and maintaining a minimal distance from them within the workplace. Therefore, this study focused mainly on nurses' presenteeism and explored its mechanism of occurrence from the perspective of head nurses. The results showed that HCP did not directly affect SNP but via SNPH indirectly affected the SNP. This study is expected to provide scientific strategies for managing nurses' presenteeism.

The results showed that the direct effect of HCP on SNP was not significant, which did not support Hypothesis 1. These findings concur with that of previous studies [11, 13]. Li argued that due to the role conflict, head nurses' cognitive preference was inconsistent with their presenteeism, particularly, anticipation preferences and benefit preferences. In addition, Shan claimed that head nurses' presenteeism promoted the incidence of SNP. Therefore, head nurses' cognitive preference possibly did not directly affect subordinates' presenteeism. Furthermore, it is probable that internal cognition is latent and impalpable, contrasting with external behaviors, which previous researchers focused on [16, 44]. Accordingly, HCP could not directly impact SNP without subordinates' observation and perception.

Moreover, this study illustrated that HCP positively influenced SNPs through SNPH, which supported Hypothesis 2. Specifically, if a head nurse is prone to identify presenteeism as a positive behavior, subordinate nurses will represent more presenteeism by perceiving this positive attitude. This result concurs with that of existing research. Li and colleagues found that although head nurses' anticipation and benefit preference were inconsistent with their presenteeism, management preference was consistent [13]. Head nurses are a trend to present management preference, and followers are also more likely to perceive leaders' management preference, as head nurses are the direct supervisor in the nursing team. Combined with the trickle-down effect of presenteeism, the management preferences of head nurses are visible and consistent with behavior, promoting subordinate nurses' perception and imitation [11].

The result verified that the mediating role of subordinate nurses' perceptions was indispensable, which implied the personal agency of followers and suggested positive outcomes of presenteeism. Specifically, junior nurses proactively exhibited work behaviors similar to head nurses' attitudes, which is in line with previous research [45]. In the work environment, employees are not passive and react in a simple manner. In contrast, employees are inclined to impact the work environment and play an important role in interpreting and reforming work conditions [46]. Furthermore, an individual's motivation to complete a variety of tasks is determined by the expectation of the possibility of task success and the values of the task [47, 48]. Thus, considering the value of showing behaviors consistent with direct leaders' attitudes is a possible reason for subordinate nurses actively molding and adjusting their work behaviors. Subordinate nurses present presenteeism as a way to manage organizational citizens' impressions. Showing similar behaviors is beneficial for obtaining head nurses' positive appraisal and support, thereby gaining leaders' appreciation and achieving promotion opportunities [49]. Within the “sanctuary” and “family” environment, nurses' presenteeism possibly contributes to showing loyalty and commitment to leaders, colleagues, and organizations [49, 50]. Meanwhile, nurses have a high workload; thus, nurses' presenteeism is likely to favor admiration from leaders and colleagues and attain positive support and organizational citizen appraisal [51]. In addition, junior nurses' presenteeism probably enhances organizational cognitive beliefs about presenteeism, shaping the organizational culture. Consequently, it contributes to organizational citizenship behaviors and promotes active organizational operations [8]. From this perspective, presenteeism is not absolutely a negative behavior. Conversely, it is possible to obtain positive evaluations, maintain performance, obtain promotion opportunities, and improve the effective functioning of healthcare departments.

### 5.2. Theoretical Implications

This study makes three theoretical contributions to the literature. First, the current research explores the effect of leaders' internal cognition, suggesting that leaders' inner cognition can also affect subordinates' work behaviors, unlike previous studies that focused on the influence of leaders' extrinsic explicit behaviors on followers. Thus, the current research compensates for the paucity of studies on the internal cognitive influence of leadership and enriches research on how leaders impact followers, which expands research on leaders' top-down influences and enriches leadership behavior theory. Second, this study emphasizes the subjective initiative of subordinates in workplaces, which breaks the traditional impression that employees are mechanical and passive receivers. Nevertheless, followers are subjective creators and are involved in bottom-up influences in the work environment. Third, this study implies that presenteeism may be related to subsequent positive consequences. This finding contradicts the previous perspective, which serves presenteeism as an absolute negative behavior, but provides valuable insights for probing into positive outcomes, broadening the boundary of presenteeism consequences.

### 5.3. Practical Implications

This study had three practical implications. First, it elucidates how head nurses impact subordinate nurses through two pathways: within-team and between-team paths. Consequently, the findings can be immensely helpful in establishing an effective intervention for preventing nurses' presenteeism; multiple interventions could be conducted, such as constructing an organizational climate of illegal presenteeism to decrease the occurrence of presenteeism. Furthermore, this study argues that leaders' attitudes and cognition toward presenteeism affect their followers' presenteeism. It is suggested that shaping and cultivating leaders' health values is an effective measure for preventing nurses' presenteeism. Moreover, the current study speculates that presenteeism among junior nurses is possibly associated with positive consequences such as obtaining leaders' appreciation and promotion opportunities. Therefore, strengthening communication between leaders and subordinates, and guiding leaders to focus on and support subordinates, may improve the transfer of negative to positive outcomes.

### 5.4. Limitations

Although this study broadens the literature on presenteeism, it does have some limitations. Subordinate nurses were required to self-report and evaluate HCPs. Furthermore, subordinate nurses' responses may not completely represent HCP but the projection of their work behavior attitude might. The current research did not separate subordinate nurses' attitudes and head nurses' attitudes, which may affect the results. Moreover, the current research simply regarded the average sum of SNPH as attitudinal consensus and team organizational climate. However, the organizational climate may be affected by objective factors, which were not measured and controlled for in the current research. Therefore, the final results may have been affected.

## 6. Conclusion

The current research confirmed that nurses are a representative occupational group with a high prevalence of presenteeism, which is related to multifaceted negative consequences. This study explored the mechanisms of nurses' presenteeism from the perspective of direct leaders. The results showed that both within and between teams, head nurses' cognitive preference toward presenteeism positively impacted SNP via subordinate nurses' perceptions. Furthermore, the results imply that junior employees have subjective initiative in the workplace, and presenteeism may lead to positive consequences.

## Figures and Tables

**Table 1:** Correlations among research variables.

	1	2	3	4	5	6	7	8	9
*Level 1*									
(1) Age	1.00								
(2) Tenure	0.060⁣^∗∗∗^	1.00							
(3) Gender	0.013	0.113⁣^∗∗∗^	1.00						
(4) Marital status	0.529⁣^∗∗^	0.514⁣^∗∗∗^	0.131⁣^∗∗∗^	1.00					
(5) Education	0.048⁣^∗^	−0.012	0.041	0.054⁣^∗^	1.00				
(6) Technical title	0.617⁣^∗∗∗^	0.610⁣^∗∗∗^	0.134⁣^∗∗^	0.490⁣^∗∗∗^	0.139⁣^∗∗∗^	1.00			
(7) SNP	0.018	0.019	0.032	0.027	0.023	0.030	1.00		
(8) SNPH	−0.012	−0.011	−0.028	−0.002	−0.018	0.010	0.196⁣^∗∗∗^	1.00	

*Level 2*									
(9). HCP	0.168	0.026	0.000	−0.002	−0.004	−0.005	0.065	0.006⁣^∗∗^	1.00

⁣^∗^*p* < 0.05.

⁣^∗∗^*p* < 0.01.

⁣^∗∗∗^*p* < 0.001.

**Table 2:** Results of multilevel path analysis.

Path	γ (SE)
a-Path	
HCP ⟶ SNPH	0.159 (0.005)⁣^∗∗^

b1-Path	
SNPH ⟶ SNP	4.364 (0.000)⁣^∗∗∗^

b2-Path	
SNPH ⟶ SNP	8.649 (0.000)⁣^∗∗∗^

Within-team indirect effects	
HCP ⟶ SNPH ⟶ SNP	0.692 (0.009)⁣^∗∗^

Between-team indirect effects (contextual effects)	
HCP ⟶ SNPH ⟶ SNP	1.372 (0.008)⁣^∗∗^

c′ (direct effect)	
HCP ⟶ SNP	−1.077 (0.332)

Total effects	
HCP ⟶ SNP	0.987 (0.487)

*Note:* HCP, head nurses cognitive preference towards presenteeism; SNPH, subordinate nurses' perception of head nurses' cognition of presenteeism.

Abbreviation: SNP, subordinate nurses' presenteeism.

⁣^∗^*p* < 0.05.

⁣^∗∗^*p* < 0.01.

⁣^∗∗∗^*p* < 0.001.

## Data Availability

The datasets generated for this study are available upon request from the corresponding author.
